# Assessing Risk Aversion From the Investor’s Point of View

**DOI:** 10.3389/fpsyg.2019.01490

**Published:** 2019-07-02

**Authors:** Antonio Díaz, Carlos Esparcia

**Affiliations:** Department of Economics and Finance, Universidad de Castilla-La Mancha, Albacete, Spain

**Keywords:** risk aversion, economic cycles, market risk premium, pricing models, big data

## Abstract

Throughout the financial literature, there is a great deal of debate about the nature of investors’ risk preferences. In an ever-changing world, the main schools of knowledge discuss the constant or dynamic basis of these preferences. Based on an exhaustive review of the subject of risk aversion, this paper contributes to filling the gap that exists in the literature on the risk aversion parameter that best fits the investors’ behavior toward risk. The main determinants of risk attitude are examined and the different and most novel methodologies and perspectives are carefully analyzed.

## Introduction

Risk aversion is one of the pillars of the theories used by economists. The theory of choice is also extensively analyzed by social sciences such as anthropology, psychology, political science, sociobiology, and sociology. Investor choice theory analyzes the behavior of individuals when confronted with the task of ranking risky alternatives and the assumption of nonsatiation. Markowitz (1952) considers that an investor is risk averse when she\he receives more utility from the actuarial value of a gamble obtained with certainty than from taking the gamble itself. Markowitz (1952) and later Tobin (1958) consider risk attitude in the portfolio selection process, implementing the theory of games and economic behavior described by Von Neumann and Morgenstern (1944). Beyond the characterization of a risk-averse utility function and how to measure a risk premium, Pratt (1964) and Arrow (1965) provide a specific definition of risk aversion. The Pratt-Arrow definition of risk aversion is useful because it provides much more insight into people’s behavior in the face of risk.

Risk preferences depend on a great deal of determinants; but, in order to make their implementation easier, the classical literature summarizes them by a single risk aversion coefficient. However, fixed risk attitude coefficients could yield strategies that do not consider the variability in the agents’ expectations. The financial literature considers risk aversion through a constant parameter or, alternatively, through a time-dependent parameter with respect to different macroeconomic and financial variables. As an additional component, recent developments take advantage of growing data processing capacity to reduce uncertainty and estimate ever more accurate changing risk aversion. The use of big data in financial markets enables faster and faster processing of data on many macro and financial variables. This better quality information provides insight into the expectations of modern investors and reduces their uncertainty about investment outcomes. In this context, the aim of this paper is to review the risk aversion literature by comparing the invariant and dynamic nature of risk preferences.

## Background

Individual preferences are complex, depending on a variety of economic, political, human, or even cultural factors. Risk aversion plays a key role to understand the behavior of different economic periods and, above all, economic recessions. This parameter amplifies the response of the most relevant macroeconomic variables to shocks of uncertainty and is, in short, the point of conciliation that makes it possible to relate finance, macroeconomics, and uncertainty. An extensive literature analyzes how fluctuations in economic uncertainty influence the dynamics of the economic cycle (see, e.g., Bernanke, 1983; Bertola and Caballero, 1994; Abel and Eberly, 1994, 1996; Caballero and Pindyck, 1996; Bloom, 2009; Bachmann and Bayer, 2013) and help explain the countercyclical behavior of major economic variables (e.g., Campbell and Taksler, 2003; Storesletten et al., 2004; Eisfeldt and Rampini, 2006; Bloom, 2009). However, the traditional way in which macroeconomists explained economic fluctuations largely ignored the importance of risk aversion in understanding economic cycles. Thereunder, the new macroeconomy recognizes that financial shocks are relevant to the real economy. Jurado et al. (2015) observe a time-varying relationship between uncertainty and real activity based on a new measure of uncertainty linking financial risk aversion coefficients with macroeconomic variables. In this sense, market risk premium and volatility reach their highest values in financial crises rather than in times of economic recession or war (Muir, 2016). Guiso et al. (2018) link changes in investor risk aversion to changes in wealth, expected income, and perceived probabilities and emotional changes in the utility function. Several authors highlight the interaction between political uncertainty and individual risk aversion. In this sense, Pástor and Veronesi (2013) incorporate this relationship into a general equilibrium model, while Brogaard and Detzel (2015) and Baker et al. (2016) examine this interaction by fitting political uncertainty through an index based on press reports.

Numerous studies show that risk aversion increases with age (e.g., Jianakoplos and Bernasek, 2006; Bucciol and Miniaci, 2011; Boyle et al., 2012; Brooks et al., 2018). Hansen et al. (1999) and Ilut and Schneider (2014) consider that consumers have pessimistic beliefs and, faced with a range of possible outcomes, act as if the worst outcomes occurred, displaying a behavior known as “ambiguity aversion.” This concept explains why many households do not invest in the stock market since the return on this investment is more ambiguous (i.e., they are not able to assign probabilities to possible outcomes) than the return on a bank deposit or a Treasury Bill (Dow and Werlang, 1992). Zhang et al. (2019) relate risk aversion with pessimism and rare disasters. Goetzmann et al. (2016) propose the availability heuristic theory in which the most recent observations have the greatest impact on an individual’s decision-making. Investors assign more probability to future stock market falls after a stock market crash. Kamstra et al. (2003) relate risk aversion to seasonal affective disorder, a depressive disorder associated with declining daylight hours. Nofsinger et al. (2018) examine the influence of behavioral biases as testosterone or stress on the individual’s risk aversion. Hoffmann and Post (2016, 2017) link up investor return experiences, confidence and investment beliefs and show why confident investors trade more than less confident investors. Falk et al. (2018) and Potrafke (2019) find a positive correlation between patience and intelligence. Suen (2018) suggests a possible discrepancy between individual and aggregate risk aversion in the context of background risk. Finally, a branch of literature proofs the influence of social factors, ethical preferences, or religious beliefs on investor’s risk attitude (e.g., Eisenhauer, 2008; Nielsen et al., 2017; Berrada et al., 2018).

### Constant Risk Aversion

Although risk preferences depend on several factors, most theoretical literature fits the risk aversion as an invariant parameter that represents the personal level of risk attitude. Simple models are very relevant as they help us set ideas. Assuming constant risk aversion allows models to reach precise and relatively simple formulas for relationships between variables. Table 1 shows some applications of constant risk aversion parameters compared to other applications with time-varying coefficients. Empirical studies show contradictory evidence for this invariable parameter over time. For instance, the risk attitude parameter appears as stable for correlative periods of time in Chou (1988), or much more unstable in French et al. (1987).

**Table 1:** Invariant vs. time-varying risk aversion applications.

Invariant risk aversion	Time-varying risk aversion
Expected utility and quadratic functions	Dependent on the economic cycle
Shape-invariant pricing kernels	Time-varying pricing kernels
Ambiguity and unawareness models	Macroeconomic and financial uncertainty measures
Point estimations for a whole data sample period	Implicit dynamic risk aversion in option prices and realized returns
	Construction of proxies of the market sentiment by PCA
	Dynamic risk aversion and the CAPM model
	Time-varying risk aversion and GARCH-M models

*Source: Compiled by the authors*.

Safra and Segal (1998) define constant risk aversion as the invariant preference relation between outcomes of two distributions when adding or multiplying them by the same positive number. Quiggin and Chambers (2004) show that risk attitude is strongly linked with the family of generalized expected utility preferences which exhibit constant risk aversion. These expected utility preferences are constant only if the investor’s utility function is quadratic, which is consistent with the capital asset pricing model (CAPM). In addition, these preferences are a generalization of both, invariant risk preferences (e.g., Quiggin and Chambers, 1998; Safra and Segal, 1998) and mean-standard deviation attitude. Other approaches link shape-invariant pricing kernels to the estimation of a constant risk aversion parameter (e.g., Lawton et al., 1972; Grith et al., 2013).

Recently, Dominiak and Tsjerengjimid (2018) generalized the preference structure in Gilboa and Schmeidler (1989) to allow for the decision maker’s *ex post* preferences to be ambiguity averse, which implies constant risk appetite. Other studies assume that investor’s risk preferences are constant and invariant to changes of unawareness and unforeseen contingencies (e.g., Karni and Vierø, 2013, 2015; Mengel et al., 2016; Ma and Schipper, 2017). Baillon and Placido (2019) demonstrate that most ambiguity models forecast that risk aversion remains constant when individuals improve overall.

### Time-Varying Risk Aversion

Considering the variability in agents’ expectations, to model the risk aversion parameter has a cost in terms of complexity. Empirical papers document time-varying risk premia in several financial markets (e.g., Fama, 1984; Hodrick and Srivastava, 1986; Keim and Stambaugh, 1986; Harvey, 1989; Li et al., 2011). There are several studies in financial literature that refer to time-varying risk aversion as a dependent parameter of different macroeconomic and financial variables. In a seminal paper on asset pricing, Campbell and Cochrane (1999) consider that an individual is more or less risk averse according to the economic and political circumstances. Their “habit formation” model incorporates large and frequent variation of the risk aversion parameter. In the same vein, Brandt and Wang (2003) develop a consumption-based asset pricing model in which aggregate risk aversion responses to both consumption growth and inflation news. Eisenbach and Schmalz (2016) consider “anxious” investors, who are more risk averse to an imminent risk than to distant one and propose a theory that leads to a downward-sloping term structure of risk premia. In the same vein, Andries et al. (2018) propose a horizon-dependent risk aversion model involving term structures of risk premium consistent with the evidence that agents are more reluctant to immediate risks than to deferred risks. Behavioral approaches have also incorporated time-varying risk aversion by way of dynamic loss aversion or conditional disappointment aversion (e.g., Barberis et al., 2001; Routledge and Zin, 2010).

As mentioned, risk preferences are closely related to economic cycles. Many studies indicate that risk aversion is countercyclical. This way, Rosenberg and Engle (2002) observe a countercyclical investor risk aversion parameter by fitting a dynamic pricing kernel. Based on the consumption-based model of Campbell and Cochrane (1999), Li (2007) shows the influence of dynamic risk aversion on asset pricing by observing that countercyclical changes in risk attitude lead to a procyclical time-varying risk premium. Furthermore, Cochrane (2017) notes that risk premia are countercyclical over time and are also coordinated across asset classes. Finally, González et al. (2018) observe the key role of time-varying risk aversion as a macroeconomic determinant of stock market betas.

Time-varying risk preferences have been modeled in several ways. A branch of the financial literature focuses on risk aversion implicit in option prices and realized returns. Option contracts offer several advantages when considering risk preferences (e.g., Bliss and Panigirtzoglou, 2004). To price options, it is only necessary to infer a discounted cash flow for a given horizon. In addition, there are options for different maturities. The multiplicity of prices for different payments on the same underlying asset provided by the options makes it possible to construct a density function for the distribution of the possible values of the underlying asset. The risk attitude implicit in option prices contains information of investors’ behavior toward risk and, hence, its variability may be captured by the jumps in risk premia implicit in the market. From option prices and realized returns on the S&P500 index, Jackwerth (2000) derives investor’s risk aversion functions and observes how shapes around financial crises change dramatically. As expected from the economic theory, these functions are positive and diminish in wealth during the pre-crisis period. On the other hand, their behavior is not consistent with the hypotheses after this event. Several authors, such as Aït-Sahalia and Lo (2000), Bedoui and Hamdi (2015), Yoon (2017), Kiesel and Rahe (2017), and Liao and Sung (2018), implement an implicit estimation of the individuals’ risk attitude from the joint observations of the cross-section of option premiums and time series of underlying assets. They examine the risk preference of market participants in different states of the world and find that risk aversion level strongly increases during stressed market conditions.

Other approaches are related to the construction of indices or proxies that represent the time-varying aggregate investor sentiment in a given financial market. The main aversion indicators can be grouped into different types: indicators that use a principal component analysis (PCA) on several financial variables (e.g., Baker and Wurgler, 2006, 2007; Han and Li, 2017; Cheema et al., 2018; Bekaert et al., 2019); indicators based on the correlation between volatilities and changes in asset prices (e.g., Kumar and Persaud, 2002); volatility indices, such as the “VIX” that uses the implied volatility of option prices on the Chicago Board Options Exchange (CBOE); and many others. For instance, Baker and Wurgler (2006) elaborate a composite index of investor sentiment derived from the first principal component of six basic proxies of investor sentiment based on various stock market indicators. On the basis of a dynamic asset pricing model with stochastic risk aversion, Bekaert et al. (2019) propose a measure of a time-varying risk aversion computed at a daily frequency that distinguishes the time variation in economic uncertainty (the amount of risk) from time variation in risk aversion (the price of risk). Most of these risk aversion indicators are used by other authors to test their ability to forecast financial crises. For example, Coudert and Gex (2008) use logit and multilogit models and observe that risk aversion indicators are good leading indicators of stock market crises.

There is a line of research linking investor risk aversion with the market risk premium derived from conditional heteroscedasticity models. The mean-variance Capital Asset Pricing Model (CAPM) of Sharpe (1964) and Lintner (1965) assumes constant second-order moments to arrive at its valuation expression, which is based on a linear relationship between expected return and risk. However, an extensive empirical evidence shows a conditional heteroscedasticity in the stock markets (e.g., Christie, 1982; Poterba and Summers, 1986; French et al., 1987)^1^. In parallel, models of Autoregressive Conditional Heteroscedasticity (ARCH) are developed (Engle, 1982) with a multitude of subsequent extensions (e.g., Bollerslev, 1986; Ding et al., 1993; Engle and Ng, 1993). Authors such as Giovannini and Jorion (1987, 1989) analyze the effects of conditional risk aversion and ARCH models for both market risk premium and performance in the static CAPM model. Given the instability of risk aversion coefficients and risk premiums over different time periods, Chou et al. (1992) improve the ARCH-in-mean (ARCH-M) model of Engle et al. (1987) with a rolling estimation procedure in which the time-varying risk aversion is integrated by a Kalman filter method. This methodology is widely applied and expanded by the use of Generalized Autoregressive Conditional Heteroskedasticity in-mean models (GARCH-M) to test the validity of dynamic risk aversion parameters in the estimation of the market risk premium (e.g., Flannery et al., 1997; Elyasiani and Mansur, 1998; Devaney, 2001; Cotter and Hanly, 2010; Dias, 2017).

Recent literature proposes text-processing techniques based on Internet search volume of certain keywords to predict returns, rather than measures based on market trading volumes and returns. There is a debate about whether these high-frequency measures actually measure time-varying risk aversion (e.g., Vlastakis and Markellos, 2012; Kearney and Liu, 2014) or whether, on the contrary, they capture retail investors’ attention toward the stock market (e.g., Da et al., 2011; Jacobs and Weber, 2012; Vlastakis and Markellos, 2012; Dimpfl and Jank, 2016) or even the investor sentiment (e.g., Da et al., 2015; Heston and Sinha, 2017). In any case, social networks can provide information on the collective behavior of investors, their state of mind, and thus allow an estimation of risk aversion at each instant of time. Financial analysis improves by increasing the speed of processing and the amount of data available. Big data and faster processors enhance investors’ forecasts of future returns. As faster and wider access to information about assets reduces uncertainty, investors tend to perceive them as “safer” (Veldkamp, 2006) and makes portfolio selection results more predictable (Kacperczyk et al., 2016; Begenau et al., 2018). Given that traditional literature states that more risk-averse investors demand more information (e.g., Willinger, 1989; Eeckhoudt and Godfroid, 2000), future research can further explore the extent to which the greater current availability of information accessible to all investors could imply a reduction in risk aversion.

## Conclusions

This literature review summarizes, critically examines, and clarifies alternative viewpoints of the most relevant contributions in each of the facets that affect the study and use of risk aversion in financial models. We review the literature with a view to providing a clear understanding of both constant or invariable risk aversion and variable risk attitude over time in the context of investor behavior and investment decisions in an environment of uncertainty.

Despite the influence of risk aversion in the investment context, most classical financial literature considers fixed values to reflect common levels of risk aversion over a full sample period. The use of a static estimate of the risk aversion coefficient over large timeframes may be desirable in simplifying models but could lead to investment decisions that do not reflect the investor’s actual attitude toward risk. On this basis, an extensive literature both in economics and in many other disciplines shows the large number of determinants of risk aversion and proofs its changing nature over time. New macroeconomy and financial theory recognize the key role of risk aversion in economic cycles, finding a countercyclical relation between risk preferences and the economic period. Many asset pricing models exhibit countercyclical risk aversion, including a behavioral dimension by way of risk-averse utility functions. Furthermore, several approaches allow the inclusion of dynamic risk aversion, such as volatility or sentiment indices, implied methods based on financial option pricing, the rolling ARCH-M model and the Kalman filter methodology, or information technology and big data analysis.

## Author Contributions

All authors listed have made a substantial, direct and intellectual contribution to the work, and approved it for publication. Corresponding author contributed to the design and implementation of the research. Both authors have collaborated in the review of the literature and in the writing of the manuscript.

### Conflict of Interest Statement

The authors declare that the research was conducted in the absence of any commercial or financial relationships that could be construed as a potential conflict of interest.

## Footnotes

^1^This evidence is one more of the traditional criticisms of CAPM, such as the unobservability of the market portfolio (Roll, 1977) or the need to incorporate additional risk factors (e.g., Banz, 1981).
